# On Nasal Vertigo

**Published:** 1887-09

**Authors:** 


### *ON NASAL VERTIGO.*

Dr. Joal, of Mont Dore, read a paper on this subject before the French Congress of Laryngology, in April, 1887, in which he stated the following conclusions:

1. There exists a nasal vertigo, a true *vertigo a naso laeso.*

2. It belongs to the group of reflex vertigos, such as gastric, laryngeal, uterine vertigo.

3. Irritation of the trigeminal filaments innervating the mucosa of the turbinated bodies, and the septum, is the starting point of the condition.

4. This irritation is transmitted to the vaso-motor nerves through the spheno-palatine ganglion, whence arises circumscribed anæmia of the brain and vertigo.

5. The affections which give origin to vertigo are (1) nasal fluxions (odors, irritant vapors, snuff, flowering grasses); (2) acute coryzas; (3) chronic catarrh, especially the hypertrophic form; (4) mucous polypi; (5) post-nasal catarrh.

6. Vertigo is especially provoked by nasal affections of little importance.

7. The nasal reflexes are principally developed in arthritic individuals.

8. Vertigo can occur alone or be accompanied by other nervous phenomena—troubles of vision, muscæ volitantes, hemicrania, nausea, vomitings, great excitability, hypochondria, intellectual disability, nightmares, spasmodic cough, dyspnœic cases, exaggerated secretions, syncope, feeble pulse, pallor of the face.

9. In order to establish a diagnosis, the nasal fossæ should be examined in every individual suffering from vertigo.

10. The recognition of nasal vertigo will sensibly diminish the number of cases of gouty, rheumatic, anæmic, congestive vertigos, as well as cerebro-cardiac neuropathy.

11. Vertigo ceases with the cure of the nasal affection to which it owes its origin. The condition has no connection whatever with Meniere's disease, and is independent of any affection of the ear. The author cites nine cases, on which, together with cases recorded by Massei, Guye, Gnuaro, Hering, Hack, and others, his essay is founded. —*Journal of Laryngology and Rhinology.*

